# The Role of Self-Improving Tutoring Systems in Fostering Pre-Service Teacher Self-Regulated Learning

**DOI:** 10.3389/frai.2021.769455

**Published:** 2022-01-03

**Authors:** Lingyun Huang, Laurel Dias, Elizabeth Nelson, Lauren Liang, Susanne P. Lajoie, Eric G. Poitras

**Affiliations:** ^1^ Educational & Counselling Psychology, McGill University, Montreal, QC, Canada; ^2^ Department of Educational Psychology, University of Utah, Salt Lake City, UT, United States; ^3^ Faculty of Computer Science, Dalhousie University, Halifax, NS, Canada

**Keywords:** network-based tutors, nBrowser, intelligent tutoring systems, self-improving tutors, preservice teachers, self-regulated learning

## Abstract

Computer-based learning environments serve as a valuable asset to help strengthen teacher preparation and preservice teacher self-regulated learning. One of the most important advantages is the opportunity to collect ambient data unobtrusively as observable indicators of cognitive, affective, metacognitive, and motivational processes that mediate learning and performance. Ambient data refers to teacher interactions with the user interface that include but are not limited to timestamped clickstream data, keystroke and navigation events, as well as document views. We review the claim that computers designed as metacognitive tools can leverage the data to serve not only teachers in attaining the aims of instruction, but also researchers in gaining insights into teacher professional development. In our presentation of this claim, we review the current state of research and development of a network-based tutoring system called nBrowser, designed to support teacher instructional planning and technology integration. Network-based tutors are self-improving systems that continually adjust instructional decision-making based on the collective behaviors of communities of learners. A large part of the artificial intelligence resides in semantic web mining, natural language processing, and network algorithms. We discuss the implications of our findings to advance research into preservice teacher self-regulated learning.

## Introduction

Technology plays a pivotal role in transforming student learning. The presence of technology in schools has increased dramatically, a trend likely to continue in the coming years. As of 2008, internet access at K-12 public schools in the United States is universal (Gray et al., 2010), while access to adequate bandwidth and connection speeds has been steadily improving. From learning how to use a word processing application to designing a website, research has shown the numerous opportunities as well as the pitfalls that access to digital devices has given students (Collins and Halverson, 2009). In general, there are many aspects of technology that can be harnessed to enhance student learning, enabling teachers to supplement traditional resources with rich, interactive multimedia content. There is a need however to better prepare preservice teachers to use technology in ways that promote active student learning (King and South, 2017). Teacher educators may lack the necessary knowledge, skills, and resources needed to effectively model technology use and integration for teacher candidates (Goktas et al., 2009). Preservice teachers often report feeling ill-prepared to use technologies effectively in lessons (Barbour and Harrison, 2016; Sang et al., 2010; Tearle and Golder, 2008) or may fail to use technology to its full potential (Lim and Khine, 2006). In using technology in the classroom, preservice teachers are prone to experience cognitive load issues that describe the cognitive capacity to represent “the number of non-automatic elaborations necessary to solve a problem.” (Salomon, 1984, p. 648). Specifically, while dealing with devices or software, particularly those unfamiliar ones, preservice teachers need efforts to monitor student attention levels, meet the needs of diverse students, assess students’ prior knowledge, as well as manage internal and external distractions. As such, teachers who are subject to high cognitive load feel difficult to respond to events and adapt their own practices (Moos and Pitton, 2014).

Research has shown that teachers who have developed a certain type of skills are better equipped to meet these cognitive demands in the workplace (Lin et al., 2005; Tillema and Kremer-Hayon, 2002; Gordon et al., 2007). As indicated, self-regulated learning (SRL) skills works that accounts for monitoring and controlling certain aspects of the cognition, affect, and behavior to attain learning goals (Zimmerman and Pons, 1986; Pintrich et al., 1993). Although beginning teachers often lack requisite knowledge or fail to deploy self-regulatory skills related to lesson planning and teaching, experienced teachers have the benefit of extensive practice and experience that typically enables efficient adaptations to changing task conditions and demands (Bembenutty, 2013; Kauffman et al., 2008; 2014; Gordon et al., 2007; Kramarski and Michalsky, 2010; Peeters et al., 2014). Kramarski (2017) suggests that teachers’ SRL have dual roles, namely, the self-regulatory processes that teachers use to plan lessons (i.e., role of a learner) and the processes they use to implement lessons in the classroom (i.e., role of a teacher). In this paper, we focus on the role of teachers as learners. Teachers who gain more knowledge related to effective self-regulatory skills are thus more likely to transfer and deploy these behaviors and skills in both situations (Kramarski and Kohen, 2015).

We review the claim that computers designed as metacognitive tools serve as a platform for teachers to gain SRL skills and also provide researchers with a mechanism to gain novel insights into development of teaching skills, such as lesson planning (Azevedo and Aleven, 2013; Lajoie and Poitras, 2017). This review centers on research and development of a network-based tutoring system called nBrowser, designed to support teacher instructional planning and technology integration. Network-based tutors are self-improving systems that continually adjust instructional decision-making based on the collective behaviors of communities of learners. In this paper, we focus on the community of preservice teachers as learners. These self-improving systems can serve as metacognitive tools by learning from users and providing supports, such as recommendations to other future system users. In nBrowser, this self-improvement is based on the convergence effect, which assumes that users’ collective behaviors will converge to a near optimal state over time similar to other crowd-sourcing networks. A large part of the artificial intelligence that allows the network to converge and provide relevant recommendations is based on semantic web mining that recognizes different features of online hypermedia documents, natural language processing that analyzes similarities among what users have written, and network algorithms that prioritize potential recommendations as the network converges. In the following section, we elaborate further on why open educational resources have been used with preservice teachers to build sophisticated understandings of lesson activities while regulating their own learning in the context of computer-based learning environments.

## Learning With Open Educational Resources

Open Educational Resources (OERs; Clements and Pawlowski, 2012) refer to any material for teaching or learning that are in the public domain or have been released under a license that allows them to be freely used, changed, or shared amongst teachers. These resources include but are not limited to instructional videos, lesson plans, online course or curricular materials made available in a software platform that allows teachers to create, modify, and share materials. We acknowledge that OERs are not the only type of resource available, however their affordances lend themselves to use with network-based tutors and they represent a wide variety of teaching and learning material. While novice teachers often lack the requisite knowledge, experienced teachers more easily retrieve mental representations without conscious effort to use them effectively in planning instruction (Berliner, 1988; Kim, 2015). Furthermore, novice teachers have high expectations—they should be able to demonstrate how planned activities are 1) aligned with curricular standards and learning objectives; 2) build on student prior knowledge and engages them in learning about the subject; 3) assess their emerging understanding and informs instruction; 4) provide feedback and adapts teaching practices to meet student needs; and 5) implement the affordances of digital technologies to support instruction (Moos and Pitton, 2014). For these reasons, this review of research has focused on lesson planning where OERs are used to facilitate less experienced teachers attempt to build a mental model represents teachers’ understandings of benefits and constraints of OERs and the impact of the use of OERs on students’ learning success (Krauskopf et al., 2015).

## Preservice Teacher Self-Regulated Learning

We conceptualize how preservice teachers learn by transforming information from OERs during instructional planning according to the (Winne and Hadwin, 1998) information-processing theory of SRL (IPT-SRL) model. Like other self-regulatory models, the IPT-SRL describes a dynamic recursive process as cognitive, metacognitive, affective and motivational dimensions unfold before, during, and after task performance (Pintrich, 2000, 2004; Winters et al., 2008; Zimmerman and Schunk 2004). Winne articulately elaborates on how he sketched essentials of IPT to self-regulation of learning in his significant work (Winne, 2001; 2018). Here, briefly speaking, SRL is processing information about learning from a lower cognitive level to a higher metalevel (Winne, 2018). From the perspectives of IPT, information is stored in the form of chunks. A schema is one kinds of chunks, which organizes information sharing similar features (Rumelhart and Norman, 1978). When a specific piece of information falls into a given schema, learners apply operations to comprehending information and therefore update or replay existing knowledge. The operation contains five processes, namely, Searching, Monitoring, Assembling, Rehearing, Translating (SMART, Winne, 2001). In SRL, learners use the information processing patterns to identify, process, and operate on information relevant to tasks (Winne, 2001; 2018). Another prominent contribution of IPT to SRL is the rule of IF-THEN form and the mechanism of control. The IF-THEN form relates to monitoring operation that compares two chunks of information and reveals how the target chunk is aligned with the features and values of the standard chunk (Winne, 2001). IF-THEN representations set the stage for operating control, allowing learners to direct their subsequent behaviors (Carver and Scheier, 1998).

The IPT-SRL model conceptualizes self-regulatory processes in terms of learner efforts to monitor and control certain aspects of learning in a recursive manner throughout four phases of defining tasks, setting goals and plans, using strategies and tactics, and making adaptations (Winne and Hadwin, 1998; Winne 2011). Each of these phases will be discussed below. As an example, we also consider a study in which a teacher implements a lesson plan featuring a MakerSpace activity. Typical of MakerSpace activities, this lesson allows students to explore their own interests and learn to use tools and materials, both physical and virtual, through a project that combine electrical circuits with digital storytelling (Nelson et al., 2020).

Definition of the Task. The initial phase of SRL begins with learners understanding demands of the task and what resources are available. Teachers may consider relevant task conditions, including OERs, time constraints for lesson planning, advice from their peers or mentors, as well as any instructional technologies and resources available. Internal factors such as prior knowledge of the domain and task, learning strategies, motivational factors, as well as beliefs and dispositions mediate teacher efforts. In defining the task, teachers may choose to review a book such as *This is Not My Hat* (Klassen et al., 2012) and judge based on their prior knowledge as well as receive advice from their peers as to whether it is suitable to the reading level of their students and the purpose of the larger activity of creating a video re-telling the story with electrical circuits. Time constraints may determine whether a teacher plans to read aloud a single copy of the story, administer multiple copies to each group of students, or show a narrated video recording of a read aloud to class in preparation for the integrated learning activity. Teachers may simply choose an alternate book with a narrative well suited for integration of circuits to re-tell the story. These internal and external conditions mediate how teachers build a mental model for the events unfolding in the planned lesson activity by defining the relevant constraints for successful implementation of the lesson.

Goals and Plans. After a learner has defined the task, the next phase consists of goal setting and planning. Setting goals and strategic planning activities involves identifying the necessary steps to accomplish successfully the lesson and establishing measurable products and timeframes. Sub-goals may also be set by learners as their outcomes may be contingent upon each other or be pursued in a given order. In the previously mentioned example, the teacher set a goal of “finding a user-friendly video recording application for tablet devices that is designed for use in elementary classrooms”. Plans to attain these goals state the sequence of actions taken by a learner, in this case the student, where each product builds on the previous action, for example “1. searching for mobile applications on Google and recording your own video; 2. taking screenshots to help students with navigation; 3. demonstrating and coaching students on how to record their own videos”. These plans may be less or more specific and be contingent on timeframes, such as one teacher’s description of “pasting the screenshots on a slide presentation *by this Frid*ay”. Evaluation standards are also an important aspect of goal setting, where learners appraise their own products to determine any discrepancies with intended results. In the case of our example lesson, teachers continuously review the instructional presentation to make adjustments to achieve their intended result. These metacognitive monitoring activities of goal setting, planning and evaluation determine whether learner attentional resources are allocated towards error correction, increased effort and information acquisition, or better time management.

Studying Tactics. To attain their own goals, learners enact different strategies to transform information. These cognitive operations can be distinguished in terms of primitive and acquired tactics and strategies to build coherent mental models. As an example, teachers may re-read and highlight segments of information in an OER that are relevant to support their instruction during the lesson’s MakerSpace activity, such as the sequence of events in the lesson. This information should be retained to reinstate it later during class. Acquired tactics are usually specific to a domain and reflect teachers’ prior knowledge, for instance, noting down in the lesson the requisite materials to complete an activity, the estimated duration of the lesson module, and inserting questions to prompt student discussions or provide instructions. An OER may fail to mention these details or might allude to them in a vague manner. Teachers who are familiar with the specific needs of different students may also adjust lesson activities to assess comprehension. Teachers’ prior knowledge is critical in identifying gaps while building mental models for the events that will unfold in the classroom, enabling them to critically appraise lesson activities and make the necessary inferences to address issues in their own understanding of a successful lesson. This demonstrates the value of relying on teacher knowledge as a factor to converge computer-based networks that support novice teachers. These tactics and strategies are also contingent upon task demands and may result in revising goals and plans based on appraisal of the outcomes through learners’ efforts to engage in metacognitive control activities.

Adaptations. The final phase consists of substantial, long-term changes evident in these self-regulatory processes as learners appraise the products of their efforts. Adaptations are different from the metacognitive monitoring activities that are manifested during learning in the form of judgements or feeling of knowing that result in strategic actions. Rather, adaptations characterize significant changes in motivational dispositions resulting from a successful lesson or a new tactic that is more likely to be enacted in future practice. In a similar manner, teachers learn from unsuccessful lesson activities, as a teacher might try an alternative sequence of lesson activities due to student confusion or group arrangement to lessen off-task behaviors. These prior experiences encoded by learners as a mental model may influence future approaches to learning. Teachers reinstate the relevant knowledge while assimilating information gained from OER to inform their efforts to plan instructional activities.

## Research Background

SRL theory conceptualizes learning as mental model construction through a process of behaviors performed on information that result in products evaluated against standards under certain conditions (Greene and Azevedo, 2007; Winne, 2017). These conditions are not necessarily conducive towards teachers attaining their learning goals. Preservice teachers may lack the prior knowledge that is necessary to automate processes involved in assimilation of information from OERs. Studies have shown that preservice teachers who lack the requisite knowledge of SRL often fail to regulate certain aspects of their own learning (Bembenutty, 2013; Kauffman et al., 2008; Kramarski and Michalsky, 2010; Peeters et al., 2014). Teachers are also expected to implement different instructional strategies and questioning techniques into lessons, continually check for student understanding, effectively utilize assessments, and create an engaging learning environment, preferably with technology. As such, a large amount of cognitive effort is required to effectively and efficiently plan lessons, integrate technology with unfamiliar devices or software, and fulfill professional expectations under short timeframes (Ertmer and Ottenbreit-Leftwich 2010; Russell et al., 2004). For these reasons, preservice teachers are prone to experience high cognitive load, which can hinder their ability to respond to events and adapt their own practices (Moos and Pitton, 2014).

Previous research has shown that teachers who have developed SRL skills are better equipped to meet the demands of the classroom (Gordon et al., 2007; Lin et al., 2005; Tillema and Kremer-Hayon, 2002) and plan instructional activities (Michalsky and Kramarski, 2008; Kramarski and Michalsky, 2010; Kramarski and Kohen, 2015). Investigating teacher SRL is a continuing concern within the field not only to reduce undesirable outcomes such as attrition and turnover, but also to gain insights into their role as external regulatory agents (Moos and Ringdal, 2012). Teachers are responsible for developing students’ abilities to engage in the same skills to become autonomous learners, and this ability has been linked to their own knowledge for how to regulate learning and their own teaching practices (White and Bembenutty, 2014; Kramarski, 2017).

We limit the scope of our review to several points drawn from studies that show the importance of preservice teacher SRL. First, SRL is critical to mental model construction during instructional planning with OERs (Azevedo et al., 2010). Second, preservice teachers can regulate successfully their own learning, but may fail to do so given both internal and external conditions, including but not limited to demands imposed by their workplace (Moos and Pitton, 2014); lack of prior knowledge that instructs them how to use technologies effectively (Mishra and Koehler, 2006); limited experience with technology (Barbour and Harrison, 2016); lack of requisite resources (Zhao and Frank, 2003); low self-efficacy (Moore-Hayes, 2011); and conflicting pedagogical beliefs (Roehrig et al., 2007). Third, task conditions may be manipulated to facilitate preservice teacher SRL by assisting them to accomplish cognitive and metacognitive tasks.

Along with the studies reviewed in this special issue, we refer the reader to a review of the literature by Kramarski (2017), where several instructional methods in a hypermedia environment are shown to design task conditions that are more favorable towards supporting teachers to engage successfully in self-regulatory processes. Namely, explicit exposure to the relevant knowledge, practice opportunities with feedback, and the use of reflective prompts and cues embedded in learning environments. The use of prompts in hypermedia environments has been shown to serve as effective scaffolds for teachers to reflect on certain aspects of their own practice (Kramarski and Michalsky, 2010). Other studies have found that reflective prompts support teacher learning by engaging in processes such as self-monitoring and evaluation strategies (Kramarski, 2008; Kramarski and Revach, 2009). This approach combined with the use of video-recorded teaching sessions where pairs of teachers alternate playing the role of a student allows them to plan, perform, and evaluate their own practice (Kohen and Kramarski, 2018). In online learning settings, the study conducted by Anderton (2006) reveals that preservice teachers are able to have increased self-regulated learning skills when they use goal analysis and evaluation and management forms. Ng (2017) uses education wiki sites as a means to foster preservice early childhood teachers’ self-regulation of learning skills, including preliminary-self-assessment of wiki sites, presentations of the wiki projects, peer assessment, revision of the projects and final-self-assessment. Findings show that the teachers demonstrated better self-regulated learning abilities when successfully involved in assessing wiki projects. The examination of collaborative discourse in online classrooms also suggests that preservice teachers can develop abilities to support student self-regulated learning by setting goals and engaging in reflections (Delfino et al., 2010; Dettori et al., 2006). The following section elaborates further on the use of computer-based learning environments as metacognitive tools for preservice teachers.

## Metacognitive Tools for Enhancing Preservice Teacher Learning

Azevedo (2005a, 2005b, 2008) outlined a metaphor to understand the role of computer-based learning environments (CBLEs) and the meta-level aspects of learning that include metacognition. The term metacognitive tool was defined in a similar manner to cognitive tool (Lajoie, 2000; Lajoie and Azevedo, 2006) as any environment that 1) assist learners to accomplish cognitive tasks by supporting cognitive processes, 2) share the cognitive load by supporting lower-level cognitive skills so that learners may focus on higher-level thinking skills, 3) allow learners to engage in cognitive activities that would be out of their reach otherwise because there may be no opportunities for participating in such tasks, and 4) allow learners to generate and test hypotheses in the context of problem solving. Metacognitive tools are different from cognitive ones in that the term acknowledges the role of CBLEs in 1) promoting learner autonomy by making decisions regarding goals and contextual conditions, 2) acknowledging the role of external regulatory agents such as peers or human tutors, and 3) modeling, prompting, and supporting behaviors that underlie information acquisition (i.e., whether cognitive, affective, metacognitive, or motivational). Broadly speaking, CBLEs designed as metacognitive tools capture and analyze key self-regulatory processes that are critical for successful learning as a means to make instructional decisions.

The focus of SRL research with preservice teacher learning over the last 2 decades has been on the means by which teachers regulate their cognition, metacognition, motivation, and engagement. Given this context, we claim that one current scientific challenge is to investigate comprehensively the effectiveness of preservice teacher SRL processes during learning from OERs by understanding relevant informational conditions in which they occur. Winne (2017) outlines several necessary conditions for comprehensive models of SRL processes in CBLEs. First, learners engage in sufficient amount of behaviors in the CBLE that can be captured by the system, such as copying and pasting relevant information from an OER into a text box. Second, that information operated on is identifiable, meaning it can be recognized as a match to the original source. Third, that the temporal deployment of each behavior is captured through a timestamp. Fourth, that the product(s) of behaviors is (are) recorded, for example the information obtained from an OER is in the final lesson plan. These conditions illustrate the need for a framework which allows researchers to understand the role of CBLEs in modeling the characteristics of how information can be operated on by the learner behaviors. We elaborate further on this notion by explaining the functioning of network-based tutors in the following section.

## Network-Based Tutors as Metacognitive Tools

Network-based tutoring systems are based on assumptions outlined below regarding the role of SRL during learning. System users are considered self-regulated learners; thus, we use the terms learner and user interchangeably to refer to anyone engaging with the CBLE. We define network-based tutors as any CBLE designed as a metacognitive tool where computational processes encode information from user interactions in the form of a node. These nodes represent information about user behaviors and are interrelated by links determined by semantic relations to create a model of user metacognitive processes. This domain model can also capture different user behaviors through distinct layers in the network, both the nature of the behavior and units of information operated on by the learner are represented in the network. Given that behaviors are deployed throughout the course of learning, a network may include properties, such as weights assigned to both nodes and links that are strengthened according to amount of user interactions. The learner model thus continually adjusts the node and link values within the network, weighing nodes more or less heavily as user behaviors are tracked by the system. A critical aspect of the artificial intelligence of the system is that links mediate node weights in the system, allowing to emulate inferences to other informational sources based on their semantic relationships (Raaijmakers and Shiffrin, 1981). For example, informational sources will be linked together according to semantic overlap and when a user interacts with one node, each node with semantic similarity will also be strengthened relative to the degree of overlap. Over time we hypothesize that the information from individual user behaviors encoded in the network will cause the weights of the network to reach an ideal state that best illustrates the amount of attentional resources allocated by learners to process that information. The term convergence effect is used here to refer to the stabilization of the weight values associated to the nodes in the network over time. This convergence enables a tutor to rely on an instructional model where instructional decision making is optimized over time, enabling the system to deliver the most suitable information to the learners. This instructional model provides scaffolds, such as resource recommendations, to learners that support their self-regulation and reach task goals. These scaffolds and supports provided to users evolve as the network is continually improved through collective behaviors made by the learners. In the following section, we elaborate further on these notions by reviewing the system components of the nBrowser system that was evaluated according to an evidence-based approach (Riconscente, Mislevy and Corrigan, 2016).

nBrowser Interface. nBrowser is designed to support preservice teacher SRL with OERs and planning instruction that implements technology in the classroom (Poitras et al., 2018a). Thus, when referring to users and learners in the context of the nBrowser system we are referring to preservice teachers. The design guidelines are based on theories of SRL and modeling principles that specify the structure of network-based tutors. The interface of the learning environment is divided into two panels organized by their different function: 1) the workspace panel where preservice teachers can seek and acquire information and make a lesson plan; and 2) the dashboard panel where preservice teachers can monitor and evaluate their own progress in attaining goals.

**FIGURE 1:**
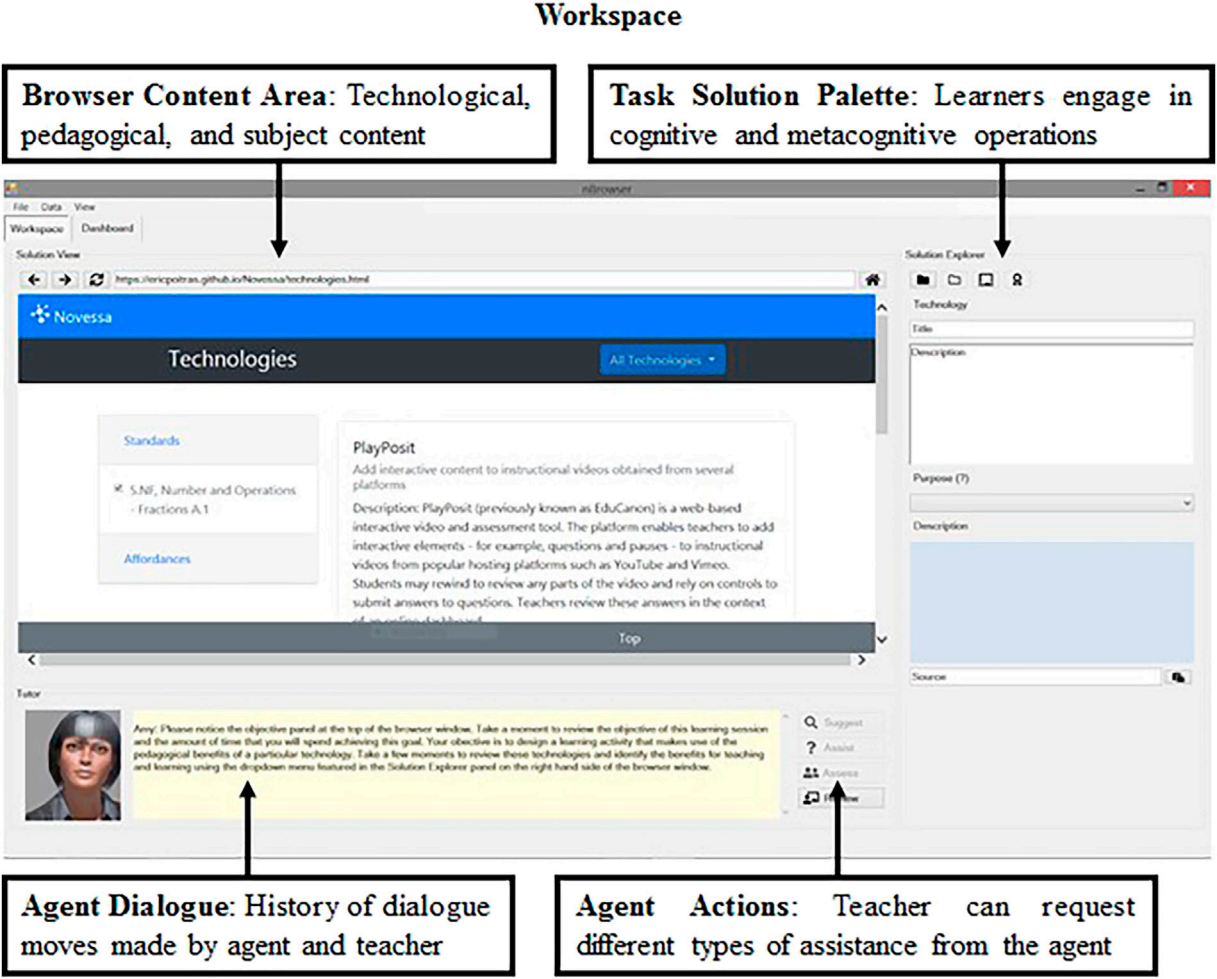
The nBrowser workspace.

The workspace, seen in Figure 1, is comprised of a browser window where the actual content of OERs (hypermedia representations of information) is presented. On the right-side of the interface, the learner transforms information using the task solution palette with functions designed to support SRL processes by breaking them down to component behaviors and making explicit the expected products of each phase. The various phases include 1) identify the affordances of digital technologies (e.g., PlayPosit, Virtual Manipulatives, Kahoot) to achieve instructional goals (e.g., present information, assess student understanding, or foster student reflection); 2) design an instructional activity while reviewing OERs; 3) analyze how and justify why the affordances of technology are aligned with the lesson design and curricular objectives; and 4) evaluate the quality of the lesson based on several standards. Preservice teachers are able to engage in each phase of SRL in a recursive manner—the system enables them to navigate to each panel to revise the relevant products after each task has been completed and reviewed by the virtual pedagogical agent. The external regulatory agent, a virtual pedagogical instructor named Amy in nBrowser shown below the main content area supports preservice teachers’ SRL in setting sub-goals related to teachers’ chosen goal, evaluating products of each phase, and delivering instructional prompts. These actions are either provided by Amy on request from the learner or provided by the system at predetermined times during task performance.

**FIGURE 2:**
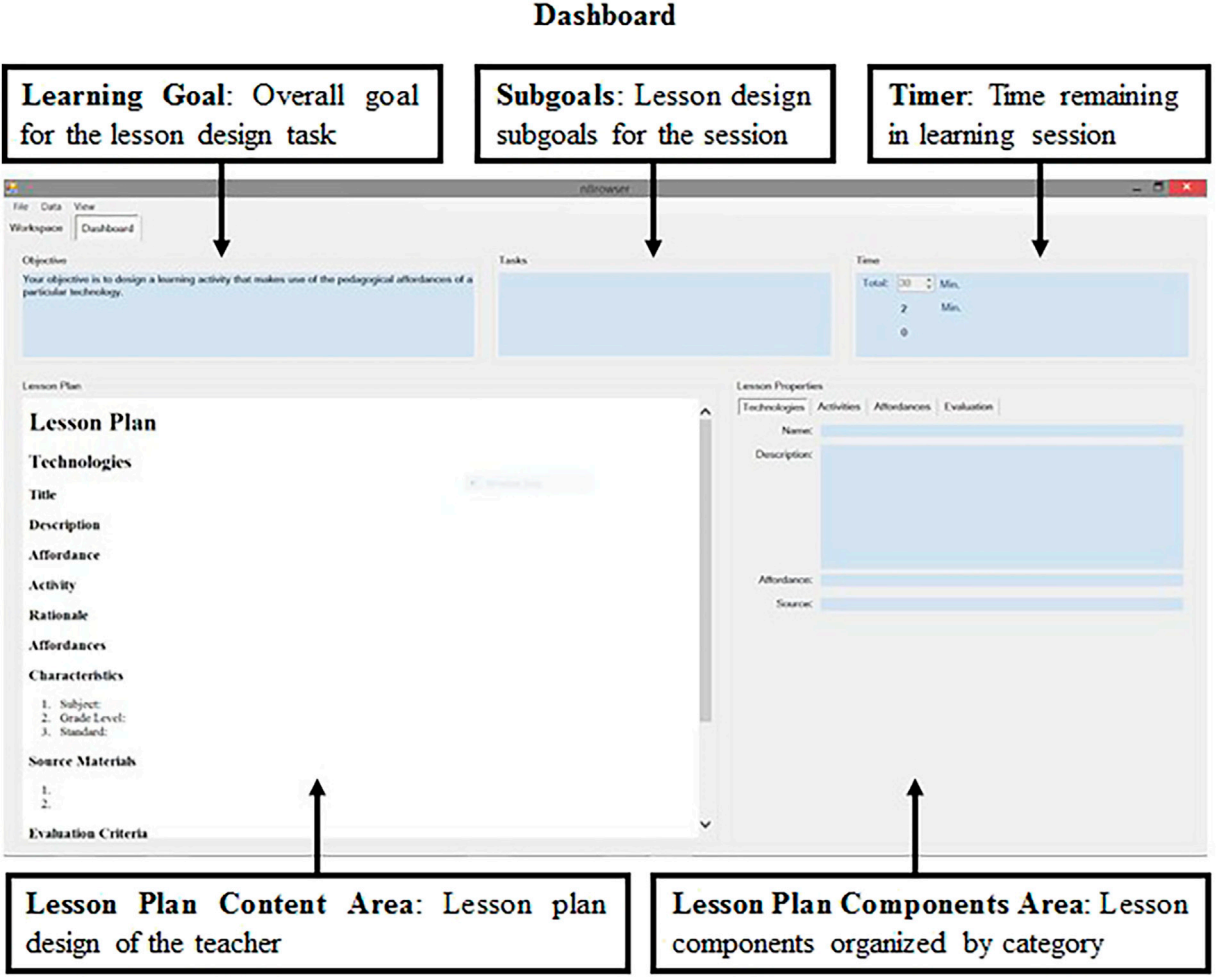
The nBrowser dashboard.

The system dashboard, seen in Figure 2, is comprised of a learning goal set by either the system designers or user (e.g., Your objective is to design a learning activity that makes use of the pedagogical affordances of a particular technology) and is related to the sub-goals set by users in the workspace. The dashboard also includes detailed plans with tasks that users can mark as completed in addition to a timer that displays the elapsed time on task. A breakdown of each lesson plan component is made available to facilitate preservice teachers’ efforts to monitor the products of their efforts. Also, the full lesson plan design is continually updated by the system and made available to users or can be saved for later use.

In summary, network-based tutors are open-ended learning environments that provide opportunities to determine what, when, and how learning occurs given the different goals and needs of specific learners (Hannafin, 1995; Land and Hill, 1997; Land, 2000). Similarly, nBrowser allows preservice teachers to be autonomous in setting goals, choosing informational sources, and using tools to create and assess task solutions. Little is known regarding the complex nature of SRL processes involved in instructional planning and technological integration that are needed to build knowledge representations of teaching that support successful technology integration (Moos and Ringdal, 2012). A critical issue centers on the contextual knowledge required to integrate subject matter, pedagogical approaches, and affordances of technologies aligned with curricular standards and student-centered learning objectives (Mishra, 2019). The system thus serves as a platform for modeling, tracking, and fostering preservice teachers’ SRL processes and utilizes a computational representation of the domain, learner, and instruction to attain these goals. We present several methodological issues and challenges that need to be addressed in terms of improving these computational representations and processes used for encoding, modeling, and fostering preservice teacher SRL.

**FIGURE 3:**
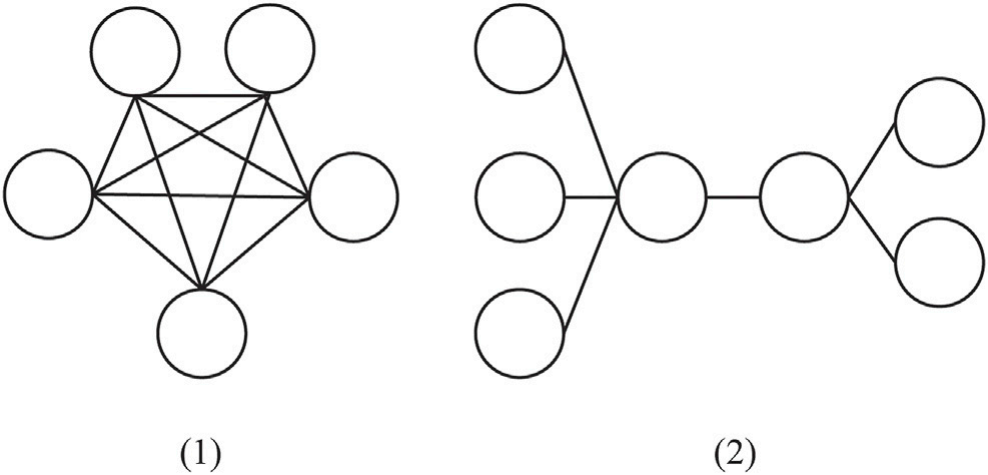
Visual representation of the main components of domain models in network-based tutors. Note. The graph **(A)** to the left illustrate a full mesh topology. The graph **(B)** to the right illustrates a partial mesh.

Domain Model. One major issue in representing information the user interacts with in the network is the arrangement of elements (i.e., nodes, links, properties, and so on). Network topology refers to the layout of a network and how nodes representing informational resources in the form of hypermedia content are connected to each other, which determines how the network converges based on user behavior. We draw a distinction between fully connected and partially connected mesh network topologies. Fully connected networks interconnect nodes representing hypermedia content (e.g., graphics, audio, video, hyperlinks, and textual information) with links spanning to every other node in the network, illustrated in the left side 1) of Figure 3. Partially connected mesh networks only contain links between some nodes based on a classification system, such as content similarity, seen on the right side 2) of Figure 3. Although this system of classification is overly simplistic, it nonetheless is useful because it allows for methodologies to author networks in a principled and scalable manner. The fully connected topology is considered more expensive than a partially connected mesh network given that the time expected for a network to reach an optimal solution is likely to increase proportionally to its size.

For instance, one classification system for partially connected mesh networks uses a systematic web browsing tool called a web crawler to find similar characteristics among hypermedia resource elements to establish subsets of nodes to link in the network. Nodes may represent either generic sources of information such as a whole OER or specific elements such as lesson modules related to a curricular standard, pedagogical approach, or grade level. Finally, nodes may be removed or included from networks in a dynamic manner using parsing algorithms to improve how the system performs its functions over time.

Domain knowledge can thus be characterized at different levels of granularity and can be differentiated in terms of relevant characteristics to the discipline. The function of any topology is to enable the system to encode user interactions with information to an associated node in the network, referred to commonly as node weight. In doing so, nodes are weighed more heavily as a result of an increasing amount of user interactions with the relevant hypermedia content detected by the system, aiming to achieve optimal node weights within the network over time that represent collective learners. The network allows for capturing multiple distinct behaviors simultaneously. For instance, users may summarize a subset of information, while highlighting hypermedia elements that mention different information and both behaviors will be encoded in the system. This allows for more nuanced instructional decision making by the system in preparation to provide scaffolded support to future users, as explained in the following section pertaining to how networks converge.

Learner Model. A large part of the artificial intelligence of network-based tutors resides in the links that connect nodes featured in the network. The key problem with learner modeling or the computational processes involved in this type of tutoring system can be summarized into two broad challenges the network faces: 1) detect user interactions with information to activate the associated node; and 2) strengthen neighboring nodes in the network based on the links connected to the active node, also referred to as spreading activation through the network. For this purpose, links are weighed in the network in a similar manner to nodes featured in the network. This property of links featured in the network is meant to represent semantic relations between the different information mentioned in OERs. It is beyond the scope of this paper to review natural language processing techniques to extract these linguistic features; we suggest McNamara, Crossley, and Roscoe (2013) for a review of the literature.

A network-based tutor can be designed to detect judgements for the relevance of informational sources for instance by allowing users to self-report a rating for the usefulness of an OER. The node associated to the OER document is then activated by the system and weighed more or less heavily on the basis of the rating value. This allows the network to learn over time which resources are the most relevant to users. Assuming the network links are weighed on the basis of the similarity of topics mentioned across OER documents, the system can infer other sources of information that may be useful to the learner. If a user judges an OER document as helpful, then similar documents might also be helpful to their efforts to seek and acquire information. Network-based tutors are able to continually update node weight values throughout the network by emulating inferences that take into account the semantic relationships indicative of topic similarity between information interacted with by learners.

We distinguish between model-driven and data-driven algorithms to spread activation throughout the network (Poitras, et al., 2019a). Model-driven algorithms are responsible for changing node weight values on the basis of other node weights of the network and user behaviors. Early examples of research have relied on the sigmoid activation function shown below to calculate the resulting node weight *N* with parameters *i* for the information associated to the node at time *t* + 1.

N(t+1,i)=N(t,i)+1(1+e−cL(t,i,j))2

**FIGURE 4:**
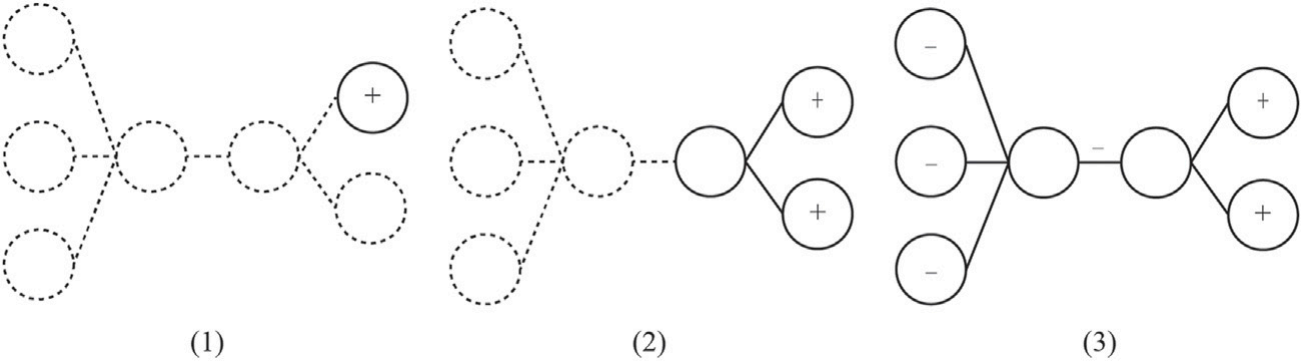
Visual representation of convergence throughout a partial mesh network. Note. The graph **(A)** shows a node activated when the system detects an SRL behavior with the relevant information, which increases the node weight property. The graph **(B)** illustrates activation spread through excitatory links (i.e., positive link weight). A node associated to a latent dimension (i.e., topic similarity) spreads activation to other related nodes, increasing the node weight property. The graph **(C)** illustrates activation spread through inhibitory links (i.e., negative link weight). A node associated to another latent dimension spreads activation to distant nodes with different topics, decreasing the node weight property.

This heuristic algorithm accounts for both the previous node weight *N* at time *t* for the same information *i.* It averages this value with the incoming activation value, represented by a sigmoid curve with equation *c* as a constant value associated to the node activation event and *L* for the link weight value with parameters *i* and *j* for both the incoming and outgoing nodes as well as *t* for time. Figure 4 illustrates how activation spreads through a network as linked node weights are strengthened using a partial mesh topology. Alternatively, data-driven models may also be implemented in the system to improve the rate of network convergence. These models are trained and evaluated on the basis of log trace data of learner behaviors to enable the system to better infer learner behaviors, as in the previous example where usefulness ratings may be inferred by the system when users fail to report them (Poitras et al., 2018b; Poitras et al., 2018c).

A serious weakness of this framework is that it assumes that learners are relatively homogenous in terms of goal setting and planning activities. In the case of preservice teachers for instance, a subset of learners might assimilate information from an OER on a grade 5 science standard pertaining to understanding features of static and current electricity. The relevant nodes should be represented through dimensions that capture the relevant metadata, including the grade level, domain, and standard under consideration. Another area for future research is the need for data-driven models to appraise the quality of products resulting from user behaviors as a means to improve network inferences. Activation spread is likely to result in suboptimal convergence without taking into account the quality of products resulting from user behaviors as well as the sequence of their occurrence in time. This imposes constraints in terms of the system interface and its ability to automate evaluations, which current research has yet to address and will be discussed further in the discussion section.

Instructional Model. Another challenge is for the system to analyze network properties in order to select and deliver instructional content that effectively supports user SRL. Past research has classified prompts as instructional scaffolds according to the specificity of information operated on by the learner and the type of behavior targeted by the prompt (Poitras et al., 2017). In terms of the specificity of a prompt, the attentional resources of a learner can be directed towards either more generic information as OERs in the form of hypermedia documents (i.e., document-level prompt) or specific parts of the document (i.e., element-level prompt) to assist preservice teachers with the SRL strategy of information seeking. Table 1 shows an example frame for such prompts using the stem “Let’s now review the following (…)”, where the system may insert in the frame a reference to the relevant information, whether a hypermedia document or element. In terms of the intended target of a prompt, the attentional resources of a learner may be prompted to process that information in a generic or specific manner. Whereas content-driven prompts highlight only the nature of the information that learners should operate on, process-driven prompts cue the users to engage in specific behaviors to assimilate information and reduce the cognitive demand of the task. This is illustrated in Table 1 using the frame “Can you (…) relevant information to add to your lesson?”, which can refer to a specific type of behavior, in this case prompting the learner to write a summary of the relevant information.

**TABLE 1:** The 2x2 framework for prompts as instructional scaffolds in network-based tutors.

	Target	
Specificity	Content	Process
Document	“Let’s now review the following (online resourc).”	“Let’s now review the following (online resource). Can you (summarize) relevant information to add to your lesson?”
Element	“Let’s now review the following (lesson activity).”	“Let’s now review the following (lesson activity). Can you (summarize) relevant information to add to your lesson?”

Note. Typical prompt sentence stems and frames denoted by “(” and “)” where information is optimized over time as the system self-improves the delivery of instructional content through the hypothesized convergence effect. The selection of instructional content is based on the analysis node properties across each layer of the network.

Preservice teachers that designed lesson plans with past versions of nBrowser had the benefit of content-driven prompts delivered by a virtual pedagogical agent, including system recommendations for OERs and prompts that guide them towards specific lesson activities within an OER. The selection of information to be recommended in the frame of the prompt is based on the network convergence, allowing the system to rely on information from collective learners indicating the most optimal resources by nodes weighted the heaviest within the network. The timing of prompt delivery by the system remains an important challenge. The nBrowser system relies on both learning events and learner behaviors, including the time spent in the learning session, the specific type of sub-goal pursued by learners, and the annotations made to the lesson. There is a need in future research to outline encoding mechanisms within the network that enables tutors to deploy prompts that coincide with the occurrence of past learner behaviors. Finally, the virtual pedagogical agent in nBrowser also supports preservice teachers through static prompts meant to 1) review completed lesson plans to identify missing components, 2) suggest hints for preservice teachers to review learning activities for instructional planning (Harris and Hofer, 2009), 3) assist preservice teachers to manage their time on task, and 4) provide example lessons where technology was implemented to support teaching and learning.

One of the main advantages of this 2 × 2 prompt framework shown in Table 1 is that the frames allow for different types of prompt taxonomies outlined in past research to be tested in the context of network-based tutors while optimizing for the content featured in the prompt frames over time. As hypothesized in the convergence effect, the network is expected to stabilize towards an ideal state as node weights are continually updated by the system as learners assimilate information to attain the same goal. For instance, a particular node may be weighted more heavily over time as users summarize the relevant information, which suggests that future users may also benefit from that same information to attain their goals. Network-based tutors can thus be designed to prompt learners to summarize the relevant information with an increasingly high likelihood as the system converges over time towards this state. The following section will elaborate further on empirical findings pertaining to the convergence effect. We will refer to our research with nBrowser to examine preservice teacher SRL with OERs by evaluating, modeling, and simulating certain aspects of this effect. Finally, we will comment on research that examined the use of content-driven prompts at the level of hypermedia elements to support preservice teacher information seeking and acquisition.

## Content-Driven Prompts at the Document-Level

According to the convergence effect, the rate at which a network is hypothesized to converge toward an optimal state is expected to increase as a function of the number of user behaviors detected by the system; and decrease according to the size of the network. If the network detects a large amount of user behaviors and the network contains a small amount of nodes, then the network is expected to converge faster toward an optimal arrangement. The key aspects of empirical research into network convergence can be listed as follows: 1) evaluating behaviors and products involved in preservice teachers’ regulation of information seeking and acquisition; 2) modeling SRL processes to detect behaviors as a means to increase the rate of network convergence; and 3) simulating the spread of activation through networks under varying conditions to ascertain the rate of network convergence. The implications of these findings are to enable the system to recommend the OERs that are most suitable to assist preservice teacher information seeking and acquisition.

Evaluating User Behaviors and Products. Initial efforts to study the convergence effect focused on evaluating the characteristics of SRL behaviors and products pertaining to information seeking and acquisition during learning and task performance. In terms of behaviors involved in evaluating the usefulness of information acquired from OERs, Poitras, Fazeli, and Mayne (2018) reported on issues related to low response rate. Preservice teachers seldom report ratings for the usefulness of online resources. Although most OERs recommended by the system were visited by students, only half of those OERs were rated by students and no more than on 3 occasions. (Poitras et al., 2018a) did confirm however that the node and link weights of the network were associated with several information seeking behaviors, including the amount of ratings for the relevant online resources, navigation events following system recommendations, and the resulting lesson plan edits. Furthermore, the node and link weights of two different networks, which were either allowed by the system to converge or not on the basis of those information seeking behaviors, were found to be significantly different. This demonstrates the ability of a network to learn from users over time and converge to support future users. Further examination suggested that a small amount of online resources rated by users was weighted more heavily in the converged network, as expected by the hypothesized effect. Although learners were found to obtain useful information more often from the converged network, these same learners also navigated to external sites to acquire information, rather than relying solely on the recommendations for OERs delivered in nBrowser. This finding suggests a need for dynamic networks where nodes are progressively added to the network as users navigate to external OERs to attain their goals.

Content-driven prompts at the level of OERs demonstrate enhanced learning and task performance by reducing the amount of attentional resources that preservice teachers allocate to information seeking behaviors. (Poitras et al., 2018a) first clustered learner behaviors into four distinct types, capturing preservice teachers’ effort to draw analogies from example lessons, plan and search for information, request hints, and evaluate the quality of their lesson designs. Although users who allocate more attentional resources to studying examples typically perform more poorly, their efforts to engage in planning, requesting hints, and monitoring were found to predict knowledge gains and design skills. Furthermore, preservice teachers who designed lessons with the benefit of the converged network reported greater gains in pedagogical knowledge than those in the control condition. (Poitras et al., 2019c) found that preservice teachers who perform better on the lesson plan design task were also more cognitively engaged in the task, reflected by use of more cognitive operations as well as a higher degree of cognitive complexity.

The recent study conducted by (Huang et al., 2021) provides an additional piece of evidence that shows the effect of preservice teachers’ SRL activities on their technology use performance. Sixty-four preservice teachers were asked to design a technology-infused lesson with nBrowser. The authors extracted six types of metacognitive activities (Azevedo and Cromley, 2004) that preservice teachers exhibited while solving the task and generated two distinct SRL profiles according to metacognitive patterns identified, namely the competent self-regulated learners and the less competent self-regulated learners. The results indicate that the competent self-regulated learners who demonstrated more efforts in metacognitive monitoring activities outperformed in terms of the lesson plan quality the less competent self-regulated learners in regulating their task solving processes. This study provides deep insights into self-regulation in CBLEs and emphasizes the pivotal role of metacognition and SRL in preservice teachers’ technology integration.

Modeling User Behaviors. Since the rate of network convergence is affected by the frequency of user behaviors, studies have trained and evaluated data-mined models to detect these latent behaviors based on overt behavioral indicators. (Poitras et al., 2018a) outlined knowledge engineered rules that allows the virtual pedagogical agent in nBrowser to prompt learners to report ratings for the usefulness of online resources. However, this approach is not only obtrusive to learning, but preservice teachers do not necessarily comply with the prompts delivered by the agent. (Poitras et al., 2018a) have shown that decision trees can successfully detect positive and negative ratings in real-time on the basis of information extracted from mouse cursor movement, position, and their combination. This concurrent model classifies the valence of user evaluation of content from the continuously-generated data stream during learning with an average accuracy of 76.3%. A retrospective model also enables the system to infer these ratings after the preservice teachers navigate from the current OER to another during learning thus enhancing the ability of the system to support future user SRL. (Poitras et al., 2018b) have shown that rule-induction methods allow the system to detect the valence of user evaluations with 69% accuracy on the basis of features that characterize panel views, count of resource navigation events, lesson plan edits, and elapsed time. These studies illustrate how ensembles of data-mined models allow the system to detect specific types of behaviors at different time intervals as a means to converge the network in a more efficient manner.

Simulating Network Convergence. Recent advances in simulated learner methods have facilitated investigation of how activation spreads throughout a network in order to optimize the convergence process. Traditionally, researchers differentiate between target and distractor nodes in the network to study under controlled conditions the time necessary for the system to converge towards the most ideal information. In a preliminary study, Poitras and Fazeli (2016) manipulated the size of a network to demonstrate how the current node-weighing scheme is more favorable than a link-weighing scheme to encode user behaviors. Poitras and Fazeli (2017) later simulated the probabilistic nature of different information seeking behaviors observed in actual human learners to demonstrate that in smaller networks, a total of 30 simulated learners were necessary for the network to converge towards a target node. (Poitras E. et al., 2018) extended these earlier findings by manipulating the amounts of nodes featured in the network and simulated learners while conducting ten replications of each simulation and varying the target node. Metrics were also established to evaluate system recommendations for target resources rather than distractors, including precision, recall, odds ratio, and F1 coefficients. These metrics allow for nuanced evaluation of the convergence process by measuring the likelihood that the system suggests target online resources (rather than distractors) and resources rated as most useful (rather than not useful). The hypothesized effect was more likely to occur under certain conditions, namely: 1) smaller sized networks (i.e., 10 nodes), as the odds are not significantly higher but remain relatively high and become increasingly variable across iterations comprising larger networks (i.e., 50–100 nodes); and 2) moderate numbers of simulated learners (i.e., 50–100 learners) where the system is most precise and sensitive in its recommendations than those obtained from larger samples (i.e., 200–500 learners). One important implication of these findings is to establish the conditions necessary for observing the effect in a laboratory setting with actual human learners.

## Content-Driven Prompts at the Element-Level

Previous studies have based their criteria for validating network topologies using the Louvain method (Poitras and Fazeli, 2016). This method is particularly useful in authoring networks with a full mesh where each node is connected to other nodes. Recent advances in domain modeling methods have facilitated investigation of less expensive solutions to authoring networks, such as the partially connected mesh network discussed earlier. As noted by (Poitras E. et al., 2019), groups of nodes may be interconnected to other nodes through a classification system that characterizes hypermedia elements in OERs in a partial mesh. For instance, a cluster of elements that mention similar topics as measured by textual overlap. The benefit of this approach is to allow an intelligent tutoring system more specific representations of information at the level of hypermedia elements in OERs.

This type of network was implemented in a study conducted by (Udy et al., 2020) to understand how intelligent systems deliver self-improving prompts. Fifty-one preservice teachers enrolled in an undergraduate course affiliated with the university’s teacher licensure education program were instructed to integrate an educational technology into a fifth grade mathematics lesson plan using a condition of nBrowser, where prompts were either made available or not. In the prompting condition, a virtual pedagogical agent in nBrowser facilitated preservice teacher information seeking and acquisition by cueing them to consider relevant information and navigating to the corresponding element in the OER. The content of prompts was also sensitive to preservice teacher’s sub-goals (i.e., to present information, assess student understanding, or foster reflective discussions) given semantic data associated to hypermedia elements in the OER. Preservice teachers could request content-driven prompts at the level of hypermedia elements many times while describing and justifying their use of technology in a lesson activity. The system guided them towards the nodes most activated in the domain model as a result of the convergence process. In the no-prompt condition, preservice teachers were not able to request prompts from the virtual pedagogical agent.

**FIGURE 5:**
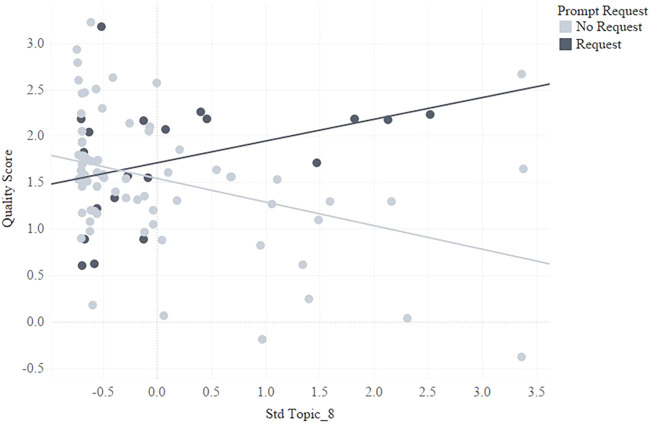
Quality of lesson plan edits across prompt request and no-request with topic mixture as a covariate.

According to the convergence effect, the network was expected to stabilize toward an ideal state allowing preservice teachers in the prompting condition to allocate less attentional resources to behaviors involved in information seeking and acquisition. The expected gains in efficiency of SRL behaviors were expected to increase the quality of the resulting products, in this case lesson plan edits. Log trace data of preservice teacher lesson plan edits with nBrowser were analyzed for topic mixtures using Latent Dirichlet Allocation as previously reported in (Vytasek et al., 2019). The quality of lesson plan edits were modeled in terms of topic mixtures in addition to time on task and the amount of characters as covariates. Using a stepwise method, these covariates were selected for inclusion for further analysis with the addition of prompt access and request as factors to examine interaction effects. Figure 5 suggests that the effectiveness of content-driven prompts that adaptively guide preservice teachers towards the most useful information in an OER is dependent upon the reason for requesting a prompt. Only users that mentioned a specific topic in their lesson plan edits also improved the quality of these edits when requesting a prompt as opposed to those that did not request a prompt.

Qualitative analysis on the four most highly rated lesson plan edits where the topic was mentioned most often revealed areas of strengths and weaknesses. The quality of the lesson plans was determined using a coding scheme based on Graham, Borup, and Smith (2012) that focuses on how teachers integrate technology, pedagogy, and content into their lesson activities. Responses focused on three important areas of knowledge for integrating technology, pedagogy, and content into their lesson activities. These three areas are 1) knowledge of content-specific instructional strategies (e.g. “T*hey will first observe a video that combines unit fractions using adults as examples. It will then continue on to a non-unit fraction example of an adult being added with a child.*”), 2) knowledge to transform content representations for teaching (e.g. “T*his will give students a visual representation of what unit fractions and non-unit fractions are and how to combine them.*”), and 3) knowledge of learner content understanding (e.g. “I *would use technology such as Kahoot for students This activity will be done after the students will learn to add and subtract fractions with unlike denominators …* ”) Although examples found individual mentions of each area, no participants included all three knowledge areas in their response. In the following section, we elaborate further on the broader implications of content-driven prompts for the design of network-based tutors to optimize instructional content.

## Implications for Future Research

The examination of the empirical evidence for the convergence effect in network-based tutors is significant in at least two major respects. First, it is evident from this research that advances in domain modeling stand to improve the ability of network-based tutoring systems to track SRL behaviors. Researchers have not treated methodologies for automated authoring of domain models in much detail. Given the amount of resources dedicated to system development, there is a pressing need for non-programmer authoring tools and infrastructure to facilitate authoring of tutors as well as crawling of hypermedia elements in OERs to build openly accessible networks in a standardized format. It will enable broader participation in advancing the modeling approach and enable the implementation of process-driven prompts that are optimized over time through fine-grained representation of informational sources. One notable example is the Cognitive Tutor Authoring Tools that allow users to create example-tracing tutors up to 4–8 times as cost effective as estimates for system development from the literature (CTAT; Aleven et al., 2016). This goal is critical for the viability and usefulness of the proposed methodology to design computers as metacognitive tools.

The second conclusion drawn from these findings is the importance of nuanced computational representations enabled by the system as the network converges. It is critical to examine issues pertaining to the validity and reliability of detections made by network-based tutors in terms of the nature of the information interacted with by learners, the type of SRL behavior, and the resulting products. There are several weaknesses to the proposed framework for learner modeling alluded to previously that can be summarized in these terms. For example, the manner in which networks can capture the time of onset for behaviors, the quality of the resulting products, and the distinct types of goals pursued by different learners. Due to these different factors, an intelligent system may optimize for information selected and delivered to the learner, while still converging towards information that although was well suited at the time, is still less suitable than an alternative that has yet to be assimilated by any learner. Further studies regarding the role of dynamic networks would also be worthwhile. Namely, a greater focus on network parsing and rendering methods to automate how nodes and links are either excluded or included within networks. These challenges drawn from the present studies provide crucial insights for further investigation to improve the efficiency of the convergence process and the capacity of network-based tutors to self-improve.

## Recommendations for Preservice Teacher Learning

Digital literacy has become a central issue for parents, educators, and policy makers, as the last 2 decades have seen a shift from a manufacturing economy towards an information economy (Porat, 1998). In the realm of education, utilizing technology in effective ways is an essential skill for both students and teachers. However, research on how teachers become more proficient in technological integration remains dependent on methodologies that too often fail to capture the processes that underlie their dual roles as learners and teachers, and the impact on students’ own learning outcomes. This paper has argued that computers can serve as both training and research platform, allowing researchers to scale measurement through unobtrusive log trace data to capture preservice teacher behaviors. Our rationale is that lesson planning imposes cognitive load during teaching and that novice teachers stand to benefit from technology-mediated instruction related to thinking and planning processes that mediate technological integration in K-12 classrooms (Moos and Pitton, 2014; Kramarski, 2017).

Future research in network-based tutors to enhance teacher integration of technology should inform best practices for the design of OERs to facilitate meaningful learning. One of the most notable directions is the use of metadata characterizing OER content to allow systems to gain insights into the semantic nature of information mentioned in hypermedia. SRL research can inform the development of schemas for semantic data that are aligned with the specific needs, interests, and knowledge of different teachers. In doing so, network-based tutors serve as a platform to self-improve instructional planning through the collective intelligence stemming from behaviors of multiple teachers. This application of crowdsourcing to the construction of knowledge in intelligent tutoring systems relies on teachers to build and modify the representation of a domain. It is also aligned with teacher preferences for new and emerging technologies used to create and sustain professional learning networks (Lautenbach and Batchelor, 2015). For this process to be sustainable, it should involve teachers directly in a transparent manner as the key stakeholders that improve network-based tutors Li et al., 2019, Ng, 2016.

## Data Availability

The original contributions presented in the study are included in the article/Supplementary Material, further inquiries can be directed to the corresponding authors.
